# Rounded leaf end modeling in Pinnacle VMAT treatment planning for fixed jaw linacs

**DOI:** 10.1120/jacmp.v17i6.6343

**Published:** 2016-11-08

**Authors:** Lori A. Young, Fei Yang, Ning Cao, Juergen Meyer

**Affiliations:** ^1^ Department of Radiation Oncology University of Washington Seattle WA USA; ^2^ Department of Radiation Oncology University of Miami Miami FL USA

**Keywords:** VMAT, multileaf collimator, rounded leaf ends, quality assurance, TG‐119

## Abstract

During volume‐modulated arc therapies (VMAT), dosimetric errors are introduced by multiple open dynamic leaf gaps that are present in fixed diaphragm linear accelerators. The purpose of this work was to develop a methodology for adjusting the rounded leaf end modeling parameters to improve out‐of‐field dose agreement in SmartArc VMAT treatment plans delivered by fixed jaw linacs where leaf gap dose is not negligible. Leaf gap doses were measured for an Elekta beam modulator linac with 0.4 cm micro‐multileaf collimators (MLC) using an A16 micro‐ionization chamber, a MatriXX ion chamber detector array, and Kodak EDR2 film dosimetry in a solid water phantom. The MLC offset and rounded end tip radius were adjusted in the Pinnacle treatment planning system (TPS) to iteratively arrive at the optimal configuration for 6 MV and 10 MV photon energies. Improvements in gamma index with a 3%/3 mm acceptance criteria and an inclusion threshold of 5% of maximum dose were measured, analyzed, and validated using an ArcCHECK diode detector array for field sizes ranging from 1.6 to 14 cm square field arcs and Task Group (TG) 119 VMAT test cases. The best results were achieved for a rounded leaf tip radius of 13 cm with a 0.1 cm MLC offset. With the optimized MLC model, measured gamma indices ranged between 99.9% and 91.7% for square field arcs with sizes between 3.6 cm and 1.6 cm, with a maximum improvement of 42.7% for the 1.6 cm square field size. Gamma indices improved up to 2.8% in TG‐119 VMAT treatment plans. Imaging and Radiation Oncology Core (IROC) credentialing of a VMAT plan with the head and neck phantom passed with a gamma index of 100%. Fine‐tune adjustments to MLC rounded leaf ends may improve patient‐specific QA pass rates and provide more accurate predictions of dose deposition to avoidance structures.

PACS number(s): 87.55.D‐, 87.55.kd, 87.55.kh

## I. INTRODUCTION

Volumetric‐modulation arc therapy (VMAT) has been shown to offer highly conformal dose distributions comparable to static intensity‐modulated radiation therapy (IMRT)^1^, ^2^ while having the advantage of faster treatment delivery^(1)^ to minimize patient motion artifacts. The implementation of VMAT pushes the requirement for greater accuracy in treatment plan modeling of the rounded leaf ends for small, irregular field sizes shaped by a multileaf collimation (MLC) system.^3^, ^4^, ^5^ This is particularly relevant because VMAT is the modality of choice for stereotactic radiation therapies where the treatment of small lesions with complex anatomies require a substantial amount of modulation to achieve adequate target conformality and the beam isocenter is not necessarily centered within the treatment planning target volume (PTV).

A VMAT plan typically employs a large number of control points (CP) with relatively small fields to achieve adequate PTV conformality. TPS models for small fields require extra effort in modeling dose profiles to match the measurements while accounting for the uncertainties and complexities associated with lateral charge particle disequilibrium, the challenges of accurately measuring dose caused by partial volume the effects of the radiation detector being used, perturbation effects for small chambers, and the spectral changes in small fields that are of less concern for equivalent square fields 5 cm or greater.^6^, ^7^, ^8^, ^9^, ^10^ In addition to electronic disequilibrium, the relative output factors for small field sizes are also impacted by the reduced phantom and head scatter, as well as attenuation of the MLC at different off‐axis positions away from machine isocenter.^(11)^ In VMAT plans, the field shapes are highly irregularly shaped small fields with dynamic leaf gaps (DLGs) that are not present in static IMRT treatment plans. The DLGs are required to prevent the leaf tips from touching as the MLCs travel through a sequence of positions specified by the TPS. Additional fine tuning of the TPS model is needed to improve the out‐of‐field dose agreement between TPS calculations and measured doses.

For VMAT treatment plans, the additional dose contributed by the DLG is included in the dose calculation of the TPS.^3^, ^4^ However, it has been shown that the TPS can underestimate actual doses by up to 20% to 40% that can add up to 2–3 Gy of unintended dose over the course of treatment.^(12)^ Better modeling of the MLC leaf gap doses is clearly needed to improve the quality of treatment plans to accurately predict in‐field and out‐of‐field doses due to opposing leaf gaps that are not backed by the jaws.

In the Pinnacle TPS (Philips Radiation Oncology Systems, Fitchburg, WI), one MLC offset table is shared by all photon energies commissioned for a particular linac. The Pinnacle TPS provides a default table as a starting point for beam modeling. During the beam model optimization process, the MLC offset table remains unchanged unless it is manually adjusted by the user. At our institution, the default MLC offset table that was applied in the original commissioned machine provided reasonably accurate out‐of‐field dose for IMRT and other static conformal photon treatment fields as determined by quality assurance measurements because the open leaf gap is covered by the backup diaphragms or jaws. However, VMAT testing with spinal SBRT fields revealed the need to increase out‐of‐field doses that were predicted by Pinnacle in order to match measured doses acquired during patient‐specific quality assurance with an ArcCHECK (Sun Nuclear Corporation, Melbourne, FL) detector array.

The purpose of this work was to develop a methodology for adjusting the rounded leaf end parameters with the aim to optimize the MLC offset table in the Pinnacle TPS to improve small field dosimetry of VMAT treatment plans and to increase the accuracy of the calculated out‐of‐field doses due to the presence of unblocked leaf gaps during treatment delivery for the Elekta Synergy‐S, beam modulator, linear accelerator (Elekta, Crawley, UK). Adjustments to the MLC rounded leaf end tables and offset parameters were accomplished by measuring leaf gap doses using three different methods followed by adjustments to the MLC leaf tip radius and offset tables to achieve better agreement between TPS calculated doses and measurements. Validation studies were completed to assess the new TPS models for improvements in QA results using the SNC ArcCHECK diode detector array.

## II. MATERIALS AND METHODS

The measurements and analyses for this study were conducted for the Philips Pinnacle TPS, version 9.6 and Elekta Synergy‐S linac commissioned for use with the 6 and 10 MV photon energies. The Synergy‐S, also known as a beam modulator linac, features a fixed jaw system with 80 interdigitating leaves that are 4 mm wide at isocenter. The maximum field size is 16 cm×21  cm at the machine isocenter that is defined by fixed diaphragms that do not move with the MLCs. The leaves are machined of a tungsten alloy with a thickness of 7.5 cm.^13^, ^14^, ^15^ The leaf ends are rounded to reduce off‐axis variations in the beam penumbra.^(14)^ The methods and procedures discussed in this paper specifically apply to additional adjustments to the rounded leaf tip leakage radius and MLC offset distance to improve the calculation of dose irradiated through the DLG that is present during VMAT delivery.

### A. Preliminary MLC quality assurance

Prior to any measurement and adjustment to the rounded leaf end model parameters, MLC calibration QA checks recommended by TG‐142 were completed.^(16)^ The Standard Imaging PipsPro MLC QA (Standard Imaging, Inc. Middletown, WI) recommended procedures were followed using the Elekta electronic portal imaging device (EPID) silicon amorphous panel to verify MLC position and calibration.^(17)^ An Elekta Direct Mount Graticule tray was placed in the gantry to mark the central axis (CAX) of the beam instead of setting up the PipsPro MLC QA phantom for the Leaf Position and Multiport tests. This eliminated the dependency of laser accuracy in conducting these tests. The EPID panel was lowered with the panel positioned so that the center crosshairs were aligned with the isocenter of the beam. The Leaf Position accuracy was verified for field widths of 4 cm, 5 cm, and 16 cm, with a length of 16 cm for all fields. These tests were performed for collimator rotations at 90°,0°, and 270°, with gantry angles set to 0° and 90°. EPID images were acquired with 5 MU delivered for each field tested. A similar procedure with the EPID imager and graticule was also applied for the Multiport test consisting of a Picket Fence test pattern using five 2 cm wide strips separated by a 1 cm space between strips. Again, MLC positioning accuracy was tested off‐axis on both sides of beam central axis. In both cases, the EPID images were analyzed using the PipsPro software with passing criteria set for ±1.0 mm.

### B. Leaf gap dose measurement

**Figure 1:**
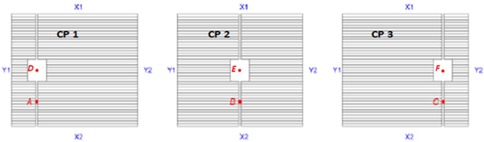
Control points irradiated with 200 MU to measure the leaf gap dose at the points A, B, and C. Dose points D, E, and F were located at the center of the open field.

Out‐of‐field dose within the leaf gap was determined by irradiating a 30×30 cm phantom comprised of Gammex 457 (Gammex, Inc., Middletown, WI) certified therapy grade solid water with three static 2.4×2.4 cm open fields delivered as three CPs in the same field, as shown in Fig. 1. Doses were measured using three different dosimetry methods: 1) Kodak EDR2 Ready Pack film (Eastman Kodak, Rochester NY), 2) Standard Imaging Exradin A16 micro‐ionization chamber (Standard Imaging Inc.), and 3) IBA‐Group MatriXX ion chamber array (IBA Group, Louvain‐La‐Neuve, Belgium). Each dosimeter was calibrated using the standard TG‐51 protocol^(18)^ for setting irradiated doses to 1 cGy/MU at the tissue maximum ratio (TMR) for a 10.4×10.4 cm field size. The standard calibration conditions for Synergy‐S at our institution are 10.4×10.4 cm square field size aligned to the machine central axis, 90 cm source‐to‐surface distance (SSD), 10 cm depth in water, and 100 cm source‐to‐axis distance (SAD). The collimator, gantry, and couch angles were all set to 0° for calibration and leaf gap dose measurements.

TG‐51 calibration output measurements on the linac were performed with a NE 2571 0.6 cc Farmer‐type ionization chamber (Phoenix Dosimetry Ltd, Berkshire, UK) and cross‐calibrated with a Standard Imaging A16 micro‐ionization chamber and a MatriXX detector array for the standard reference conditions as previously described. A Keithley, Model 35614EBS (SN 19218) electrometer (Tektronics, Inc., Beaverton, OR) was used for all measurements conducted in this study. Kodak EDR2 film calibration was also conducted by irradiating separate sheets of film with a 10.4×10.4 cm field size at a depth of 10 cm depth in solid water, aligned to 100 cm SAD on central axis (CAX) of the linac. Published work evaluating the use of EDR2 film for IMRT quality assurance have shown minimal field size related changes in optical density (OD) response for square fields between 2 and 14 cm in size.^19^, ^20^, ^21^ Monitor units (MU) were calculated and delivered under standard conditions for 20, 50, 100, and 150 cGy to determine irradiated dose based on the optical density (OD) of the film. The doses used for the film irradiations were confirmed by ionization chamber (IC) measurements with the NE 2571 under standard calibration conditions in solid water. The film was scanned in transparency mode on an Epson Perfection V750 Pro scanner (Epson America, Inc., Long Beach, CA) to create Joint Photographic Experts Group (JPEG) image files that were imported into ImageJ^(22)^ for analysis. The intensity of the dose profile pixels along the central axis of the irradiated field and 4 cm on each side were averaged and correlated against the known calibrated dose. A least squares curve‐fit of optical density to delivered dose was calculated to determine the correlation of film dose to OD that is given in Eq. (1):

Dose [cGy]=65.284 ln(OD)+88.598

Upon completion of preliminary MLC and dosimetry QA, point‐dose measurements were made in 30×30 cm solid water with special chamber slab for the A16 ion chamber. A step‐and‐shoot (SNS) field having three CPs with a 2.4×2.4 cm square centered vertically was manually created with a 1 mm leaf gap on either side of the treatment field as shown in Fig. 1. The ion chamber was placed at a depth of 10 cm in solid water and physically centered within the 2.4×2.4 cm squares and within the leaf gap for each control CP as shown in Fig. 1. The chamber measurement in the 2.4×2.4 cm field was made to ensure that adjustments to the rounded leaf end model parameters would not affect dose to the targeted dose point for small open fields. Six data points were collected for both 6 MV and 10 MV photon energies with 200 MUs delivered to each CP. Each measurement was repeated three times and the final recorded dose was a calculated average over the three readings. The same measurements were repeated for the MatriXX detector array and EDR2 film for both 6 MV and 10 MV photon beams. The point doses acquired from the MatriXX detector array were recorded and compared to the dose‐point measurements acquired with the A16 ion chamber. The EDR2 film was converted into dose profiles using the ImageJ software where the image intensity per pixel was converted into dose using Eq. (1) for comparison with the doses acquired by ion chamber and detector array.

### C. Rounded leaf end optimization

Treatment plans were recreated in Pinnacle under the same irradiation conditions as the measurement. Dose points were calculated at the center of each open field and centered in the 3.0 mm leaf gap for each CP. The Pinnacle dose calculations were compared with the A16 ion chamber measurements, dose points generated by the IBA‐Group OmniPro Software (Version 1.7) and EDR2 film dosimetry for both 6 MV and 10 MV photon beams.

The Pinnacle rounded leaf end parameters are shared by both photon energies and it was necessary to find a compromise between the two energies. Since there are no automodeling scripts with the Physics module in Pinnacle, it was necessary to iteratively change the MLC offset and rounded leaf tip radius values manually, recompute the dose points, and evaluate how well the dose points agreed with the measurements.

The general strategy applied was to adjust the ratio of the center to off‐axis dose first using the MLC offsets, then shift all doses at all three locations to match the ion chamber measurements by adjusting the rounded leaf end radius. The MLC offsets were adjusted first because the observed discrepancy between the A16 IC measured leaf gap doses and the Pinnacle calculations was greatest in the difference between the center and off‐axis dose points. Small MLC offset adjustments in increments of 0.01 cm led to relatively large changes in center dose to off‐axis dose ratios. For example, from an offset of 0.07 cm to 0.08 cm, the dose ratio at center to off‐axis is 1.73, this ratio decreased to 1.56 with an additional +0.01 cm MLC offset. Fine tuning of these gross adjustments were accomplished using the rounded leaf end radius parameter.

**Figure 2:**
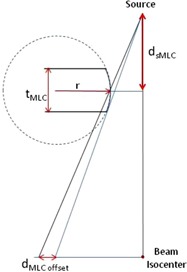
The rounded leaf tip radius, r, and MLC offset distance, $d_{\text{MLC}\;\text{offset}}$, were adjusted to improve the in and out‐of‐field DLG dose calculation. The distance from the source to the center of the MLC which defines r is $d_{\text{MLC}}$.

The MLC offset is defined as the difference in MLC position as measured by the nominal optical light field and the projected physical leaf edge at 100 cm SAD.^23^, ^24^ These values estimate the transmission increase in the rounded leaf tip relative to transmission for the full MLC thickness^(24)^ as shown in Fig. 2. Using the Pinnacle default rounded leaf tip leakage radius (12.2 cm) and default MLC offset values as a starting point, the MLC offset was incrementally and iteratively shifted by 0.01 cm. To shift the MLC offset, the offset value (such as 0.01) is added to each value in the offset column of the Rounded Leaf End Specification Table. The percentage difference in leaf gap doses were calculated between the Pinnacle calculated dose points off‐axis and at the center and corresponding measurements. Subsequent offsets were selected to minimize the calculated and measured point dose differences at positions A, B, and C shown in Fig. 1. The objective was to adjust the MLC offsets until ratios determined by the Pinnacle TPS agreed with A19 IC measurements to within 5%.

MLC offset in the positive (+) direction had the overall effect of lowering the leaf gap doses with larger decreases observed to the center of the field compared to the off‐axis leaf gaps. A negative (‐) shift raised all computed leaf gap doses with relatively higher leaf gap doses at the center of the field with lower increases to the leaf gap doses off‐axis.

After the dose ratio between the center and offset gaps was adjusted, the rounded leaf tip radius was used to increase or decrease the overall Pinnacle calculated doses for all gap locations. Increasing the rounded leaf tip radius decreased transmission, thus lowering the leaf gap doses at the center of the field to a slightly lesser amount than the off‐axis dose. Decreasing the rounded leaf tip radius had the opposite effect of increasing the overall transmission through the leaf gap. For example, a decrease in the radius by 1.0 cm increased the leaf gap dose by on average 1.5 cGy and 1.9 cGy at the center and off‐axis gaps, respectively, for 6 MV photons. Slightly higher values were observed for 10 MV photons where these values were 3 cGy at the center and 4.8 cGy at the off‐axis leaf gaps.

The 6 MV photons were optimized first to minimize dose differences between IC leaf gap measurements and Pinnacle dose point calculations. Further adjustments were made with the 10 MV photons so the point dose discrepancies were also minimized. The iterative optimization trial process ended when adjustments to the rounded leaf tip parameters did not lower the dose point differences any further for both beam energies.

### D. Pinnacle dosimetric verification

After the MLC modeling parameters were optimized using static fields, it was necessary to assess the out‐of‐field dosimetric improvement for arc delivery. Verification tests were conducted using a series of square field with sizes ranging from a 2.4 cm to 16 cm, with a leaf gap manually created in the field using Pinnacle with a collimator rotation of 20° to minimize interleaf leakage. Dose profiles for the 360° arcs were measured and analyzed using an ArcCHECK Phantom System accompanied by SNC Patient Software (Version 6.2.3; Sun Nuclear). The beam data were collected with the ArcCHECK phantom set up at the linac radiation isocenter. The gamma factor was calculated and the dose measurements were compared against calculated profiles. The gamma factor for 3% dose difference, 3 mm distance to agreement, and an inclusion threshold of 10% maximum dose was evaluated for each field size. The two‐tailed Wilcoxon signed‐rank test was applied to paired sets of gamma scores to examine the significance of statistical differences between the original and improved TPS models for square fields less than 4 cm in size.

Additional dose profile comparison between the original and optimized MLC models was conducted for plans generated using the phantom and volume contours sets recommended by TG‐119.^(25)^ A similar procedure was performed where gamma factors were computed using the 3%/3 mm, and 5.0% dose threshold criteria in evaluating the agreement of the measured dose profiles against the calculated treatment plan. Gamma factors calculations for the 2%/2 mm criteria was also evaluated because this analysis is more sensitive to dose changes resulting from MLC model adjustments to dose contributed by the open leaf gap both in and out of the VMAT treatment field.

End‐to‐end testing using the standard IROC head and neck phantom protocol for VMAT credentialing was completed for the Radiation Therapy Oncology Group (RTOG) 1205 protocol. The IROC results before and after the Pinnacle TPS model changes were compared to assess improvements in dosimetry by an external reviewer.

## III. RESULTS

### A. Measured leaf gap dose

A summary of the measured doses in the 2.4×2.4 cm irradiated area and through the open, static leaf gap are provided in Table 1. Within the irradiated treatment area, the A16 ion chamber, MatriXX, and EDR2 film dose measurements were on average within 2% of the uncorrected Pinnacle calculated doses for 6 MV photons and closer to 3% for the 10 MV photons.

**Figure 3:**
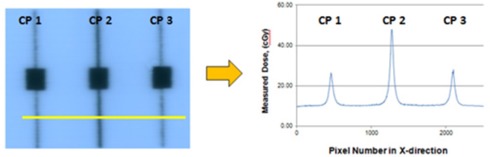
Kodak EDR2 film dose profile measuring leaf gap dose for 6 MV photons on an Elekta Synergy‐S linac.

Greater measurement uncertainties were observed in the leaf gap region of the field where increased variations in dose were observed between dosimeters. Because the MatriXX ion chambers are spaced 7.62 mm apart, the doses between detectors are interpolated. Film dosimetry measurements, as shown in Fig. 3, provided the best resolution of all dosimeters evaluated, but these results indicated that the doses through the leaf gap varied between 59% and 65% higher than the uncorrected Pinnacle calculated dose at Point B in Fig. 1. The A16 ion chamber dose measurement estimates ranged between 7% and 10% higher for the same dose point. Doses measured along the leaf gap were much less consistent. Based on previous feedback our institution received on past RTOG credentialing for SBRT with the IROC spine phantom, the A16 ion chamber results were more reasonable and appropriate for optimizing the MLC beam modeling parameters in Pinnacle.

**Table 1:** Summary of point doses measured and calculated in the treatment field and out‐of‐field leaf gap as labeled in Fig. 1 for CP1 (left), CP2 (center), CP3 (right)

	2.4×2.4 cm *Center of Field*					*Static MLC Gap*				
*Energy (MV)*	*Dose Point*	*A16 Ion Chamber (cGy)*	*MatriXX Meas. (cGy)*	*EDR2 Film (cGy)*	*Pinnacle Calc. (cGy)*	*Dose Point*	*A16 Ion Chamber (cGy)*	*MatriXX Meas. (cGy)*	*EDR2 Film (cGy)*	*Pinnacle Calc. (cGy)*
	D	131.9	131.1	131.5	134.7	A	30.6	21.7	30.2	30.2
6	E	131.8	130.3	133.5	132.8	B	37.1	34.5	55.8	33.5
	F	134.3	131.4	135.6	135.1	C	21.1	22.0	27.2	30.9
	D	145.0	142.5	148.6	146	A	35.3	24.2	35.8	30.1
10	E	141.4	139.7	144.2	142.6	B	37.1	40.6	58.4	34.5
	F	143.9	142.2	146.4	146.6	C	24.1	26.1	35.8	30.8

Measured doses are higher for the leaf gap formed by CP2 at the center of the field compared to the off‐axis CPs in all three measurement modalities, as shown in Fig. 3. The film dose at CP2 is greater than the averaged off‐axis CPs by a factor of 1.9 and 1.6 for the 6 MV and 10 MV photon beams, respectively, on film. This gives the appearance that the leaf gap width is nearly twice as wide at the center when indeed the leaf gap widths are 1 mm a distance of 39 cm from the source^(15)^ for all three CPs. The gaps for CP1 and CP3 appear narrower because the phantom is irradiated from an off‐axis angle and some of the dose is attenuated along the curved tip of the MLC. CP2 is defined by the MLC tip and is not attenuated by any part of the MLC so a higher dose would be anticipated. However, there appears to be larger uncertainties associated with the EDR2 leaf gap measurements when compared to the A16 and MatriXX results.

### B. MLC model optimization

During initial VMAT quality assurance testing of TG‐119 phantom cases, we found that the default beam modulator rounded leaf end tables, MLC tip leakage radius of 12.2 cm and offset of 0.0 cm, did not produce sufficient agreement with ArcCHECK QA results of the same plan with a minimum passing criteria of 90% for a gamma factor of 3%/3 mm. Instead, the MLC default parameters for the Elekta Synergy linacs with standard 1 cm MLCs provided a better fit and they were used in our initial commissioning stages for VMAT where the leaf tip radius is 15.0 cm with a 0.1 cm MLC offset.

Following a series of MLC offset shifts and evaluation of dose differences between measured and calculated leaf gap point doses, we found that adjustments to the MLC rounded leaf end modeling parameters in Pinnacle did not have any effect on the point doses calculated at the center of the irradiated 2.4 cm square fields where the percent difference between the maximum and minimum calculated dose were 1.2% and 0.5%, respectively, for 6 MV and 10 MV photon beams. These values were calculated for all trials that were evaluated over the entire course of this study.

**Figure 4:**
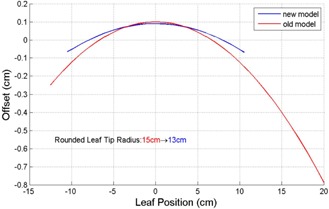
Leaf offset and rounded leaf tip radius of the old and new model.

Beam modulator MLC model parameters provided better results than the parameters implemented during VMAT commissioning by increasing the rounded leaf tip radius from 12.2 cm to 13.0 cm with a +0.1 cm MLC offset. This corresponds to a decrease in rounded leaf tip radius of 2.0 cm from the 15.0 cm default MLC table for the 1.0 cm MLC parameters. Figure 4 shows the graphical difference between the initial MLC offset table and final MLC rounded leaf offset table used in Pinnacle as a result of this study.

Because both 6 MV and 10 MV photon energies are shared by the same MLC offset table, the final solution is an averaged compromise between both energies. It was impossible to match the measured leaf gap dose for one energy without observing larger dose discrepancies in the other energy. Initially, the Pinnacle leaf gap doses were 1.4% less than the IC measurements off‐axis and 10.9% less at the center for 6 MV photons. Higher discrepancies were observed for the 10 MV photons where the Pinnacle doses were less than the measured doses by 17.2% off‐axis and 21.8% at the center. Following the optimization procedure, the Pinnacle calculated leaf gap doses were 30.1 cGy off‐axes (1.6% off‐axis discrepancy) and 39.4 cGy (6.2% higher than the IC measurement) at CAX for the 6 MV photon leaf gap doses. The 10 MV photons were not as well matched, but the dose discrepancies were reduced from 14.7% to 12.5% off‐axis (30.9 cGy) and 17.9% to 5.5% (39.7 cGy) along CAX.

### C. VMAT beam model verification

**Figure 5:**
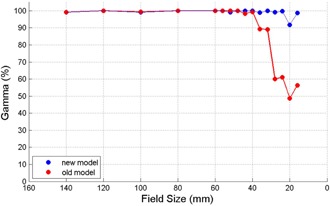
Comparison of ArcCHECK QA agreement between the old and new models for MLC‐collimated treatment fields.

Verification tests with a single 360° arc delivering dose from a fixed square field with manually created leaf gaps shows distinct improvement in gamma scores when the ArcCHECK measured profiles were compared against the dose calculations for the original and MLC rounded leaf end optimized TPS models. For VMAT fields with square field sizes less than 4 cm, the QA gamma scores from the MLC improved model were significantly different from those measured using the initial TPS model (p=0.03). The median gamma scores were 99.1% and 60.6% for the new and original TPS models, respectively. No significant differences were observed in square fields 4 cm or greater. As seen in Fig. 5, with the new model gamma reaches 99.0%, 99.9%, 99.2%, 99.7%, 91.7%, and 98.8% for fields with size of 3.6 cm, 3.2 cm, 2.8 cm, 2.4 cm, 2.0 cm, and 1.6 cm, whereas with the initial model gamma for the same fields are, respectively, 89.3%, 89.1%, 60.1%, 61.1%, 48.6%, and 56.1%.

These results show that larger field sizes pass the gamma test for TPS agreement consistently close to 99% or better possibly because the proportion of the dose contributed in the treated volume is affected by the penumbra region much less for larger fields than smaller ones and the leaf gap lengths are also considerably shorter. The 80%‐20% penumbra for the Elekta beam modulator at the rounded leaf ends is typically between 3.1 and 5.3 mm.^(14)^ For small field sizes, the penumbra and dosimetric effects of the MLC rounded leaf ends play a much greater role in the dose calculation. Therefore, MLCs that are fine‐tuned to match leaf gap doses pass at much higher gamma 3%/3 mm rates for small, as well as large, field sizes.

Commissioning quality assurance measurements with the revised rounded leaf end model revealed improvements from 0.8% to 3.6% in gamma index for a passing criteria of 3%/3 mm distance to agreement and applying a 5% minimum dose threshold to include the analysis of dose points outside the primary treatment area, as summarized in Table 2. Higher gamma‐index pass rates for the 2%/2 mm criteria were observed particularly for the 10 MV photons where an 8% improvement was observed for the H&N case and 7% for the multitarget cylinder VMAT plan. While the gamma index is primarily an indicator of in‐field agreement of delivered doses to TPS calculations, examination and analysis of point dose profiles along the superior and inferior edges were used to evaluate out‐of‐field impacts. The out of field doses predicted by the TPS were still underestimated compared to the doses measured by the ion chambers in the MatriXX detector array; however, they were reduced to a more tolerable average of approximately 1% to 2%.

**Table 2:** ArcCHECK results of rounded leaf end quality assurance improvements for gamma index of 3%/3 mm and 2%/2 mm with a minimum 5.0% isodose threshold, using three case studies recommended by the TG‐119 report

*TG‐119 Case Study*	*Energy (MV)*	*Gamma* 3%/3 mm *Initial (%)*	*Gamma* 3%/3 mm, *MLC Adjusted (%)*	3%/3 mm *Gamma Index Improvement (%)*	*Gamma* 2%/2 mm *Initial (%)*	*Gamma* 2%/2 mm, *MLC Adjusted (%)*	2%/2 mm *Gamma Index Improvement (%)*
Head & Neck		97	97.9	0.9	92.5	94.2	1.7
Multitarget Cylinders	6	97.5	98.3	0.8	91.1	93.9	2.8
C‐Shape		95.9	96.7	0.8	89.3	91.4	2.1
Head & Neck		95.3	98.6	3.3	81.5	897	8.2
Multitarget Cylinders	10	95.1	96.8	1.7	79.0	86.1	7.1
C‐Shape		82.7	85.5	2.8	66.5	69.9	3.4

**Figure 6:**
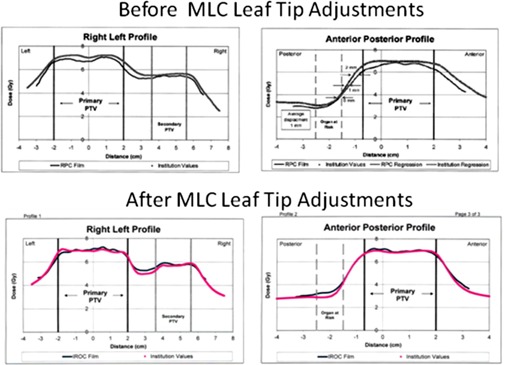
IROC film dosimetry compared to TPS dose calculations during end‐to‐end tests with the IMRT/VMAT H&N phantom. Improved dose agreement was observed for TPS profiles generated after the MLC rounded leaf end model parameters were optimized.

The new TPS model was applied to end‐to‐end VMAT dosimetry testing with an IROC head and neck (H&N) phantom that is commonly used for credentialing an institution to conduct RTOG studies. The test results of the H&N phantom irradiated using VMAT fields generated from the revised Pinnacle model demonstrated better agreement between the IROC measured dose profiles on film versus our institution's reported values compared to the original beam models as shown in Fig. 6. Although both original and new TPS models passed IROC performance criteria, the thermoluminescence dosimeter (TLD) point‐dose measurements were improved with dose differences between measurement and TPS predictions being 1% to 2% compared to corresponding values of 3% to 5% from the original set of measurements. Film dosimetry for the MLC adjusted model also passed with 100% of the measured dose points being within 7% of the TPS doses and within 4 mm distance to agreement. For a gamma index criteria of 3%/3 mm, the passing rate was 97.4% as measured with a SNC ArcCHECK phantom and software.

## IV. DISCUSSION

The Elekta Synergy‐S linear accelerator was designed to deliver stereotactic radiation therapy with relatively small fields. In VMAT treatments, the collimator is rotated between 10° to 25° to minimize interleaf leakage while optimizing spatial resolution.^(26)^ To mitigate a potential collision of the rounded leaf ends during arc delivery, a physical leaf gap is required. For the Elekta Synergy‐S, a 1.0 mm leaf gap is located 39 cm from the target in the gantry, for all activated dynamic leaves during arc therapy delivery.^(14)^ The beam modulator is the only Elekta MLC system that has a DLG that is not blocked by backup jaws during VMAT delivery. This condition leads to greater inaccuracies in out‐of‐field irradiation doses to the patient compared to other Elekta linacs, particularly if the default offset and rounded leaf end tables are not carefully adjusted in the TPS.^3^, ^4^ Multiple static field (step‐and‐shoot) IMRT treatments are not subject to this effect because all leaf gaps are positioned under the fixed jaw during irradiation.

This study demonstrates a need for a novel method for refining MLC rounded leaf TPS model parameters and validating the results to improve the predicted dose contribution due to the open DLG present during VMAT delivery on an Elekta linac with fixed jaw, beam modulator system. Validation tests showed substantial improvement in gamma 3%/3 mm QA results with a maximum of up to more than 40% for a 1.6 cm square field. The result of this work improved the predicted accuracy of the out‐of‐field doses particularly in small, irregularly shaped field with an off‐axis beam isocenter. The originally commissioned TPS underestimated out‐of‐fields doses by about 5%. Although this is a relatively small amount, others have reported even greater discrepancies^(12)^ that could negatively impact treatment outcomes due to higher‐than‐expected doses to critical avoidance structures.

The concept of MLC rounded leaf modeling in the Pinnacle TPS has been available since the release of Version 7.4.^5^, ^24^, ^27^ A number of studies have been published on the calibration and quality assurance of MLC rounded leaf end and DLGs for commissioning IMRT and VMAT TPS.^(4,12,24,27–38)^ Many of these publications are particularly applicable for use with Varian linear accelerators that use the Eclipse TPS. In this case, the dose contribution is adjusted by modifying the DLG that is adjusted during linac commissioning to account for differences in the radiation versus light projected field.^33^, ^36^ Others have developed methods for calibrating and commissioning the rounded leaf ends of the MLCs with the Pinnacle TPS for Elekta linacs with asymmetric jaws,^5^, ^34^, ^39^, ^40^ but they do not specifically address leaf gap doses that are not blocked by jaws as in the case for the Elekta beam modulator. This work addresses the lack of a strategy to deal with VMAT dosimetric errors that can be corrected by measuring the leaf gap dose and adjusting the MLC offset distance and rounded leaf tip leakage radius.

The primary weakness of this work lies in the uncertainty of the true leaf gap dose as indicated by the differences in measured point doses among the three methods discussed. Measuring absolute dose through the leaf gap is prone to errors and uncertainties caused by the detector size, differences in energy spectrum, lack of charged particle equilibrium, and the potential overresponse of the A16 detector stem comprised of a high‐Z material at low photon energies.^(41)^ Although the MatriXX detector array provides more dose point measurements in a single irradiation and is efficient to use, it is subject to more uncertainties in the leaf gap region because of volume averaging effects in each of the ion chambers (4.5 mm diameter, $0.08\;{\text{cm}}^{3}$ effective volume) and computational interpolation of the dose between detectors.

The overresponse of Kodak EDR2 film in the leaf gap that is not present in the 2.4 cm irradiated square field is caused by the film becoming oversensitive to scattered radiation in low‐dose regions.^(24)^ High‐Z materials, such as silver bromide, in Kodak EDR2 film also causes a higher probability for photoelectric events to occur that results in an energy dependent overresponse in leaf gap film dosimetry compared to actual doses delivered in water.^(42)^ Others have observed similar results during MLC calibration and commissioning with EDR2 film of Picket Fence images.^24^, ^43^ While EDR2 film is an excellent method of visually inspecting the MLCs for leaf position calibration, it is not a reliable method for determining irradiated dose to the leaf gap.

The A16 micro‐ionization chamber has an outer diameter of 3.4 mm and internal collecting volume of $0.007\;{\text{cm}}^{3}$. This dosimeter is less prone to volume averaging effects unlike the chambers within the MatriXX detector array. Measurements acquired with the A16 ion chamber seem to produce results that were closest to the unmodified TPS calculations and were used for as the benchmark doses to achieve during the optimization process. Other leaf gap measurements were useful in confirming that the leaf gap doses at the center of the field were indeed higher than the doses measured off‐axis on both sides.

Better agreement may have been achieved, but an important limitation of the Pinnacle TPS was the lack of multiple MLC leaf tip offset table for all energies that are commissioned. Although MLC rounded leaf end parameters could be initially optimized to provide dose calculations that were closer to the 6 MV photon measured data, they did not match as well with the 10 MV photons. Additional adjustments produced better results for the 10 MV photons, but the dosimetry improvement was also accompanied by a worsening of agreement with the 6 MV measured data. It would be ideal if separate tables were available for fine‐tuning adjustments of all beam energies.

Manual optimization of the MLC rounded leaf end parameters is a tedious process because MLC modeling in the Pinnacle Physics Module is limited to interleaf leakage through the tongue‐and‐groove interface between MLCs and transmission through the leaf thickness. The MLC rounded leaf end machine settings reside outside the beam modeling optimization module and are not included in any of the beam automodeling scripts programmed within Pinnacle. The verification of any improvements or worsening of the beam model is also performed manually in the treatment planning module of the Pinnacle TPS. Additional development of software tools is needed to overcome these limitations.

The MLC rounded leaf edge table provided in this work may be used as a substitute for the default tables provided with the Pinnacle TPS for other clinics using the Elekta beam modulator linac. No factory settings were changed for beam matching with other machines in the department. However, due to the lack of shielding in the treatment vault, the beam modulator was manufactured for 6 and 10 MV photons only. The Elekta flattening filter for the 10 MV photons on the Synergy‐S linacs is slightly different for machines manufactured to deliver 6, 10, and 18 MV photons. Caution should be used in applying the results presented in this publication without performing the recommended point‐dose measurements and verifying that further adjustments are not necessary.

## V. CONCLUSIONS

The work presented provides a methodology for improving the dose calculation of transmitted irradiated doses through MLCs with rounded leaf ends as they travel along the full width of an Elekta Synergy‐S beam modulator linac. Because there are not many institutions using the 4 mm microleaf system in the United States, the literature is lacking in publications recommending additional MLC adjustments needed for VMAT commissioning or improving overall dose model calculation versus actual beam delivery quality assurance agreement. In addition to the Elekta Synergy‐S linac, the methods and results presented here may also apply to any fixed diaphragm (jaw) system having an unblocked leaf gap during VMAT treatment delivery using the Pinnacle TPS. Without these adjustments, the default MLC parameters loaded in the Pinnacle sample machines may lead to inaccurate underestimates of dose outside the treatment field even though the overall in‐field doses are acceptable enough to pass usual quality assurance measurements. Implementing these procedures clinically will not only improve patient‐specific QA pass rates, but it may also improve the overall quality of treatment plans, with more accurate predictions of dose deposition to critical avoidance structures adjacent to the planning target volume.

## COPYRIGHT

This work is licensed under a Creative Commons Attribution 3.0 Unported License.
